# The Impact of Color Vision Deficiency on the Capability of Ophthalmologists to Diagnose Benign and Malignant Choroidal Tumors

**DOI:** 10.3390/jcm12072744

**Published:** 2023-04-06

**Authors:** Mutasem Elfalah, Saif Aldeen AlRyalat, Nakhleh E. Abu-Yaghi, Mona Mohammad, Ibrahim AlNawiaseh, Deema Rayyan, Moath Albliwi, Mohammad Elfalah, Fawaz AlSarairah, Yacoub A. Yousef

**Affiliations:** 1 Division of Ophthalmology, Department of Special Surgery, School of Medicine, The University of Jordan, Amman 11942, Jordan; 2 Department of Surgery (Ophthalmology), King Hussein Cancer Center, Amman 11941, Jordan; 3 Ophthalmology Department, Faculty of Medicine, Mutah University, Mutah 61710, Jordan

**Keywords:** color blindness, fundus, nevus, malignant, color vision deficiency, protanopia, deuteranopia

## Abstract

Background: Color vision deficiency (CVD) is an under-reported problem among medical personnel, and its impact is still not well characterized. We aim to assess the impact of CVD among ophthalmologists on the accuracy of diagnosing different benign and malignant choroidal lesions. Methods: This is a cross-sectional study conducted on ophthalmologists. We used a web-based survey to collect responses through professional ophthalmology society social media. The survey included a set of five images for normal fundus, choroidal nevus, circumscribed choroidal hemangioma, choroidal metastasis, and choroidal melanoma, wherein each image simulated the three main types of CVD: protanopia, deuteranopia, and tritanopia, in addition to a non-simulated image. Results: Forty-one participants were included, with a mean age of 40 (±9.2) years. They were 28 (68%) men and 13 (32%) women. Participants showed significantly low accuracy for definite diagnosis for circumscribed choroidal hemangioma, nevus, melanoma, and metastasis when the images simulated protanopia and deuteranopia, but not tritanopia. Nevertheless, participants maintained the capability to recognize the nature of the lesions for both simulated and non-simulated images if they were benign or malignant, thereby ensuring immediate referral for specialized care. The exception was with simulated choroidal nevi images, wherein participants incorrectly assigned simulated protanopia and deuteranopia nevi images to malignant lesions. Conclusion: Protanopia and deuteranopia affected the accuracy of diagnosing several choroidal lesions; however, ophthalmologists with those two simulated CVDs were still able to discriminate between benign and malignant tumors.

## 1. Introduction

Color vision deficiency (CVD) refers to the inability to distinguish certain shades of color. The estimated prevalence of this problem in the general population has been reported to be up to 8% among males (0.4% among females), with similar figures estimated for medical personnel [1,2].

There are several types of CVD that can be either acquired or congenital. Anomalous trichromacy refers to an abnormality in the alignment of one of the three cones that result in reduced sensitivity to a particular color. If the sensitivity to red is reduced, it is termed protanomaly; if the sensitivity to green is reduced, deuteranomaly; and if the sensitivity to blue is reduced, tritanomaly. Dichromacy occurs when there are only two functioning retinal cones that perceive color with the absence of one cone type. The three types of dichromacy are protanopia, deuteranopia, and tritanopia. Finally, monochromacy refers to having no cones or only one type of cone, which results in no color perception [3]. Despite its relatively high prevalence, many people may be living with the condition while being completely unaware of it.

In ophthalmic practice, the diagnosis of choroidal tumors is primarily clinical without the need for pathologic confirmation. The ocular oncologist relies mainly on clinical features including the color of the lesion to diagnose these lesions. Melanoma is generally a thick melanotic choroidal lesion, while circumscribed hemangioma is orange in color, and metastasis appears creamy yellow. Posterior uveal melanomas are often seen as a raised, dome-shaped lesion with a gray-brown color and uneven margins. Occasionally, a melanoma may lack pigmentation. Other possible causes of choroidal lesions include a benign or suspicious nevus, hemorrhagic macular degeneration, metastasis, hemangioma, hamartoma of the retina or retinal pigment epithelium, diffuse melanocytic proliferation, or detachment of the pigment epithelium, retina, or choroid. Ciliary body tumors that can resemble a melanoma include iridociliary epithelial cysts, intraocular foreign body granulomas, melanocytic nevi, melanocytomas, leiomyomas, Fuchs adenomas, sarcoid nodules, and metastatic tumors. During the evaluation of a suspicious choroidal lesion, various clinical characteristics such as color, thickness, subretinal fluid, orange pigments, drusen, halos, and others should be examined. Color is an important diagnostic characteristic of choroidal tumors, with melanomas usually exhibiting shades of brown, gray, black, or white, and haemangiomas often appearing orange-red. Most metastases appear white, except for renal and carcinoid secondaries, which may appear orange [4,5,6,7,8,9,10,11,12,13,14,15].

Therefore, we theorize that ophthalmologists who have problems defining colors correctly may fail to diagnose these conditions correctly, and this may cause delayed referral and treatment, and consequently serious morbidity and mortality for patients. The effect of having CVD on the accuracy of differentiating and diagnosing benign and malignant fundus lesions among ophthalmologists has never been studied before; therefore, the aim of this study is to assess the impact of simulated CVD on the accuracy of diagnosing different benign and malignant choroidal lesions among ophthalmologists.

## 2. Methods

### 2.1. Study Design and Participants

This is a cross-sectional study conducted on ophthalmologists from Jordan. We obtained institutional review board approval from the King Hussein Cancer Center (22 KHCC 008), and participants gave consent regarding their participation. We conducted this research in concordance with the latest Helsinki declaration. We used a web-based survey to collect responses. We distributed the survey through professional ophthalmology society social media, wherein we targeted Jordanian general ophthalmologists, retinal specialists, and ocular oncologists.

### 2.2. CVD Simulation and Assessment

**Figure 1:**
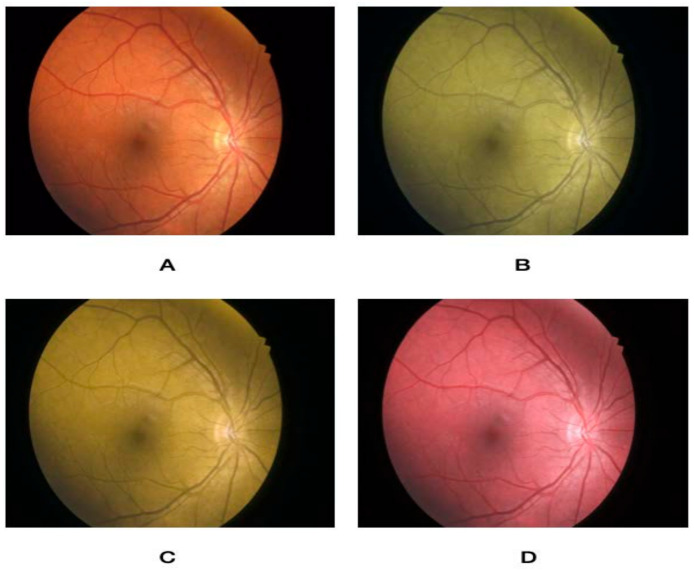
Fundus images for normal fundus (**A**), under simulated protanopia (**B**), deuteranopia (**C**), and tritanopia (**D**).

**Figure 2:**
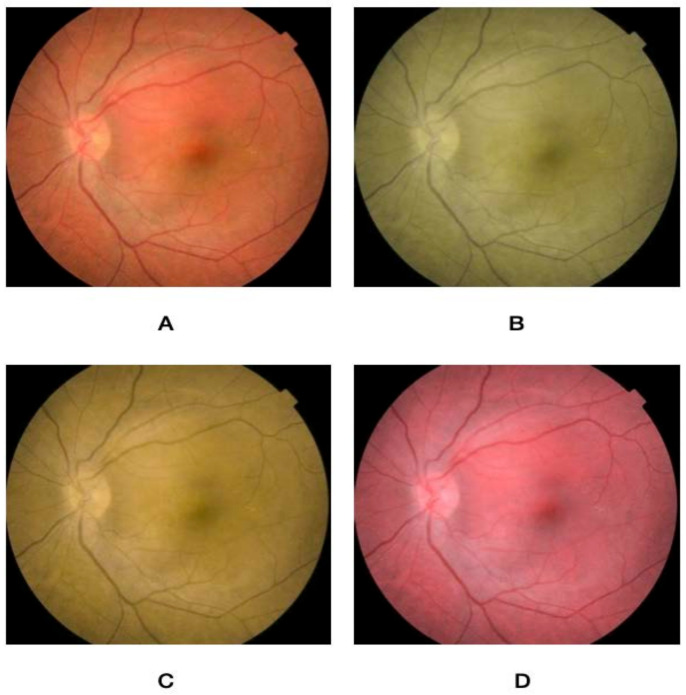
Fundus images for circumscribed choroidal hemangioma (**A**), under simulated protanopia (**B**), deuteranopia (**C**), and tritanopia (**D**).

**Figure 3:**
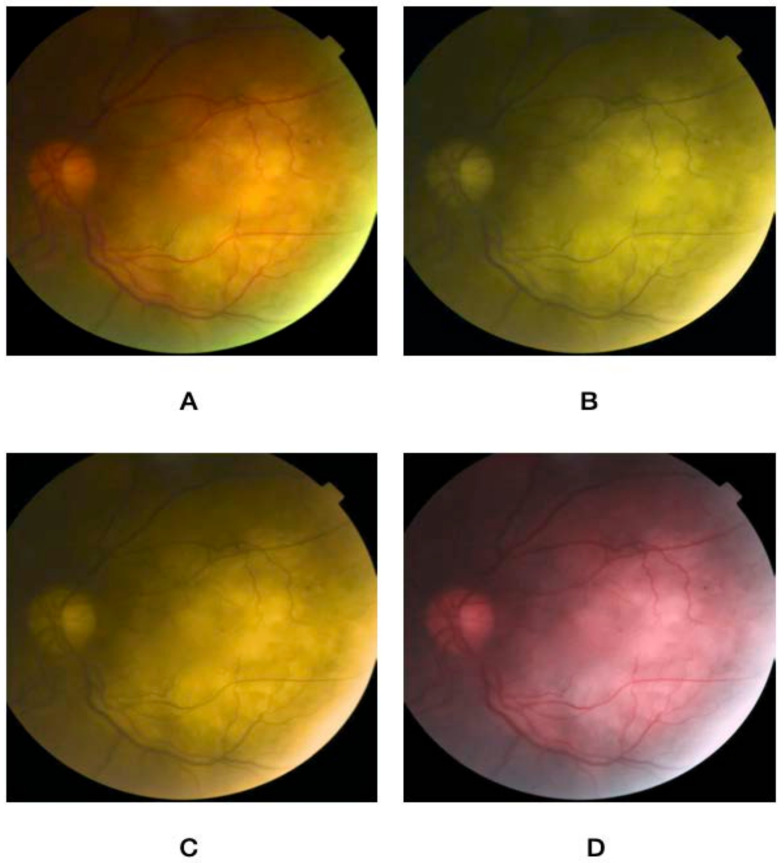
Fundus images for choroidal metastasis (**A**), under simulated protanopia (**B**), deuteranopia (**C**), and tritanopia (**D**).

**Figure 4:**
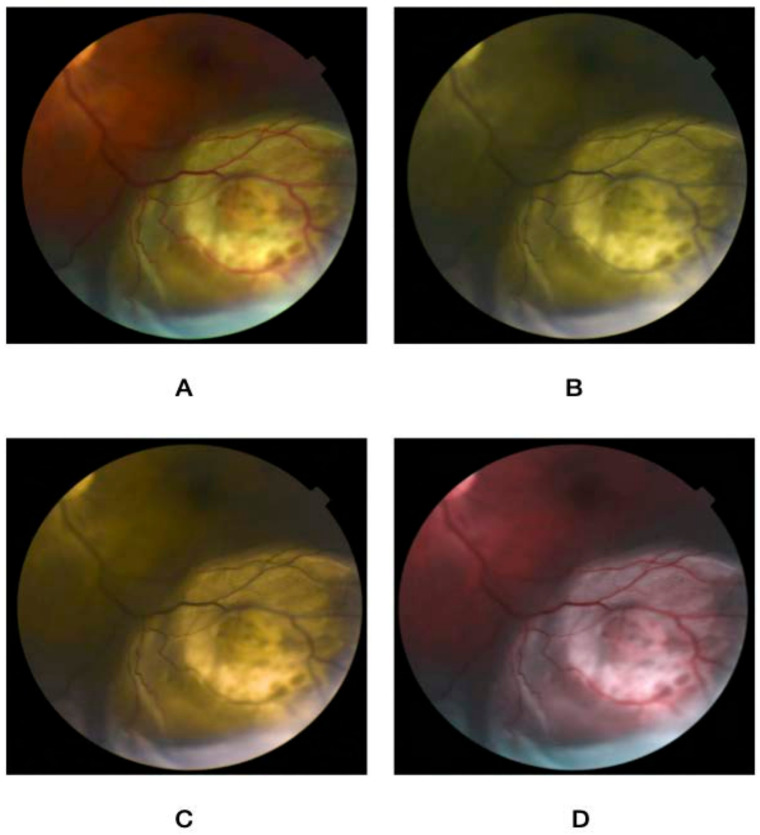
Fundus images for malignant melanoma (**A**), under simulated protanopia (**B**), deuteranopia (**C**), and tritanopia (**D**).

**Figure 5:**
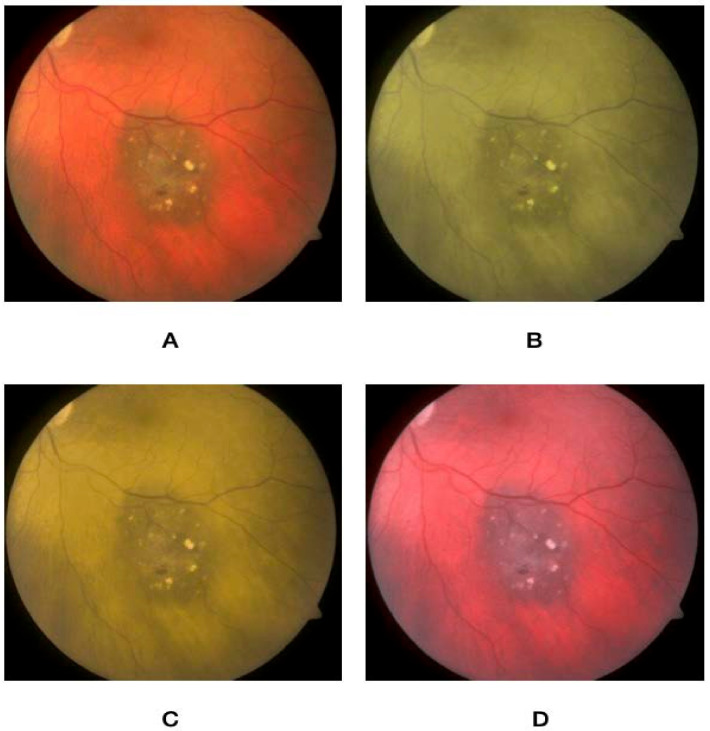
Fundus images for choroidal nevus (**A**), under simulated protanopia (**B**), deuteranopia (**C**), and tritanopia (**D**).

Using an online form, the nature of the project was described, and participants signed up to partake in the study. The rater was first asked to take an online color vision deficiency test to confirm the absence of preexisting CVD. The questionnaire then asked about their demographic variables (i.e., age, gender, and occupation), in addition to specialty and years of practice. This was followed by a brief overview of the characteristics of choroidal nevus, circumscribed hemangioma, metastasis, and melanoma. After that, a set of five images for normal fundus, choroidal nevus, circumscribed choroidal hemangioma, choroidal metastasis, and choroidal melanoma were presented. Images were extracted from the database of the King Hussein Cancer Center. Each image was then altered to simulate the three main types of color vision deficiency, namely protanopia, deuteranopia, and tritanopia, and were stored in addition to a non-simulated image. Figure 1, Figure 2, Figure 3, Figure 4 and Figure 5 show fundus images for normal fundus, circumscribed hemangioma, metastasis, malignant melanoma, and nevus along with their simulated protanopia, deuteranopia, and tritanopia variants.

Twenty fundus images with these different benign and malignant lesions were randomly distributed representing four sets of five: simulated protanopia images, simulated deuteranopia images, simulated tritanopia images, and non-simulated images. Participants were requested to answer two main multiple-choice questions for each image (a total of 20 images for each participant); one asked about the definite diagnosis of the disease and the other asked if the lesion was benign (and does not need an immediate referral to an ocular oncologist) or malignant (and needs immediate referral). Benign images included a normal fundus image, choroidal nevus, and circumscribed choroidal hemangioma. Malignant images included choroidal melanoma and choroidal metastasis. Thereafter, participants were considered to have a passing score in a set if they correctly answered the questions for at least three out of the five images in that set. All participants who failed to correctly diagnose the normal fundus photo in a non-simulated image were excluded from this study.

To transform fundus images into what a protanope, deuteranope, and tritanope ophthalmologist can see, we used the Vischeck color blindness simulator in Fiji software [16], which has been proved to be highly accurate in simulating colorblind images [17].

### 2.3. Statistical Analysis

We used SPSS version 26.0 (Chicago, IL, USA) in our analysis. We used mean (±standard deviation) to describe continuous variables. We used count (frequency) to describe other nominal variables. We used Fischer exact tests to analyze the difference between the number of correctly diagnosed images between specialties. All underlying assumptions were met. We adopted a *p*-value of 0.05 as a significant threshold.

## 3. Results

A total of 41 participants were included in this study, with a mean age of 40 (±9.2) years. They were 28 (68%) men and 13 (32%) women. Two-thirds were general ophthalmologists 28 (68%), while 13 (32%) were retinal specialists or ocular oncologists. The average number of years in practice for included participants was 11.2 (±7.6) years, ranging from one year to 35 years.

All participants correctly diagnosed normal images when non-simulated. However, participants had significantly low accuracy for definite diagnosis for normal fundus images as well as for circumscribed hemangioma, nevus, melanoma, and metastasis when the images simulated protanopia and deuteranopia, which was not the case for tritanopia (Table 1). On the other hand, participants could correctly decide if the lesions for both simulated and non-simulated images were benign or malignant, thus mandating referral for specialized care. The exception was in the nevi simulated images, where participants incorrectly assigned simulated protanopia and deuteranopia images for nevi as “needs referral”, with p values of 0.007 for simulated protanopia and 0.003 for simulated deuteranopia images (Table 1).

When we evaluated the overall score for the questionnaire for each set of images (considering correct answers for at least 3 out of the 5 questions as a passing score) for diagnosis of intraocular tumors, we found that participants with simulated protanopia and deuteranopia images had a significantly higher score deciding whether the lesion was benign or malignant compared to giving a correct definite diagnosis. This indicates good ability to realize the nature of the lesion (benign vs. malignant) but limited capability to recognize the exact pathology behind each image in this group with CVD (Table 2). There was no significant difference between general ophthalmologists versus ocular oncologists and retina specialists regarding definite diagnosis or discriminating capability between benign and malignant lesions (Table 2), even though ocular oncologists and retinal specialists showed relatively better scores for definite diagnosis with simulated protanopia and deuteranopia images when compared to general ophthalmologists.

## 4. Discussion

Many aspects of modern-day life necessitate accurate color vision, and these can range from simple everyday tasks to more complex color-dependent functions. This study aims to assess the impact of CVD on the accuracy of diagnosing different choroidal lesions, using a high-quality simulation on a diverse group of ophthalmologists. We found that ophthalmologists with simulated protanopia and deuteranopia are not able to correctly diagnose the exact pathology in patients with benign and malignant choroidal tumors, even though they still have the capability to recognize whether the choroidal lesion was a benign lesion that needs observation or a malignant lesion that mandates referral to an ocular oncologist for immediate treatment. Color blindness did not affect the capability of experts in eye tumors (ocular oncologists and retinal specialists) to correctly diagnose the exact pathology in the choroid; however, general ophthalmologists had a more dramatic decrease in their capability of diagnosing the pathology of different choroidal tumors correctly.

The results of our current study go hand-in-hand with a recently published study that assessed the impact of CVD on diabetic retinopathy staging, wherein graders with color vision deficiency had lower staging accuracy, a difference that was more pronounced among protanopic graders [18].

The increasing evidence in the literature points towards the need for prevocational screening for CVD in applicants, especially for specialties or subspecialties that require good color discrimination. Among doctors and healthcare workers, 5% of dichromats and 25% of anomalous trichromats were reported to be unaware of their colorblindness [19]. As a result, this may lead to clinical implications and significantly hinder their ability to accurately diagnose or stage certain diseases. In fact, a previous study on CVD that assessed the responses of physicians to accurately identify certain clinical signs through colored photographs showed that they were less likely to correctly identify the specified clinical signs and were less confident in their responses in comparison to the control group with normal color vision [20]. A colorblind ophthalmologist also described difficulties in diagnosing fundus pathologies, wherein lesions that are normally pigmented as red appeared as blue, including the red ophthalmoscopic reflex [21]. Another challenge that may be faced among colorblind ophthalmologists during retinal examination is that of distinguishing artifacts (e.g., melanin pigment) from other disease manifestations (e.g., retinal hemorrhages), as these lesions may have a similar appearance upon examination [22]. This would lead to falsely reporting a higher stage than the actual stage. Finally, differentiating retinal hemorrhages and other manifestations in patients with chorioretinal degeneration, wherein the background of the retina appears to be darker than normal, poses an additional difficulty [22]. These limitations further portray the crucial importance of correct color identification on clinical practice, diagnosis, and staging of eye diseases.

Color serves as a crucial diagnostic characteristic of choroidal tumors. Melanomas typically exhibit shades of brown, gray, black, or white, whereas hemangiomas are commonly orange-red. Most metastases appear white, except for renal and carcinoid secondaries, which can appear orange. Although digital cameras, such as the widefield camera, are extensively used for screening and apply various algorithms to recreate fundus colors, the resulting images may not reflect reality and may mislead clinicians. Therefore, tumors should be examined ophthalmoscopically instead of solely relying on photographs [6,7,8,9,10,11,12].

Correct identification of colors is essential for certain careers and requires screening prior to job placement. Although some medical schools in the UK and all in Taiwan have implemented testing for colorblindness as a prerequisite for medical school, it is not a requirement in the majority of medical schools globally [23]. Screening can test for the severity of colorblindness, provide counseling, and help individuals choose career paths that do not require accurate color identification. Although the impact of medical errors resulting from colorblindness on patient care is complex to establish, it is strongly recommended for patient safety. While clinical appearance is not the sole factor in diagnosing choroidal tumors, various techniques such as fundus examination, ultrasound, angiography, and OCT may improve the diagnostic accuracy of colorblind ophthalmologists. Although our study showed limited ability of participants with colorblindness to diagnose choroidal tumors based on clinical images, they can still distinguish benign from malignant lesions, thus avoiding harm to patients. Utilizing other diagnostic tools is expected to enhance their tumor-diagnosis abilities, although this aspect was not analyzed in our study.

This study had many limitations that should be considered in future research. We were limited in the number of cases we could include in the survey, as we wanted to keep it from becoming tedious for participants to complete; therefore, we chose clear-cut diagnostic cases that would not cause confusion for general ophthalmologists in order to accurately gauge the capabilities of colorblind participants. Furthermore, the participants knew that the only photos they would have to evaluate were ones showing either a normal fundus or a classic choroidal nevus, a classic circumscribed choroidal hemangioma, a classic choroidal melanoma, or a classic metastatic choroidal tumor, and that their “diagnostic accuracy” would be expected to be substantially higher than in real life where the differential diagnosis should be wider as it includes other choroidal tumors such as amelanotic choroidal nevi, borderline melanocytic choroidal tumors, choroidal neurilemomas, choroidal osteomas, retinal astrocytomas, subretinal infiltrates of primary vitreoretinal lymphoma, and a variety of fundus lesions that can simulate a malignant or benign fundus neoplasm (e.g., subretinal hematoma, localized suprachoroidal hematoma, solitary hypertrophy of the retinal pigment epithelium, inflammatory chorioretinal granuloma, and fundus lesions of sclerochoroidal calcification). Therefore, we suggest that future studies consider evaluating smaller amelanotic lesions and other lesions in the differential diagnosis to be closer to real life. One more limitation inherent in the design of this report is that while we showcase a lower diagnostic accuracy for images simulating CVD, in fact, fundus lesions are usually distinguished based on size, color, consistency, and supporting features. Supporting features might be drusen, orange pigments, and calcifications, among others. Future studies should focus on further exploring the value of such features on diagnostic accuracy. Moreover, it is also worth mentioning that the role of participants’ experience was not found to be a significant contributor, and this finding should be further explored in future studies.

## 5. Conclusions

CVD can affect an ophthalmologist’s ability to diagnose fundus pathologies. We conclude that simulated protanopia and deuteranopia CVD affected the accuracy of diagnosing several fundus lesions, including circumscribed hemangioma, choroidal nevus, choroidal melanoma and metastasis, and even normal fundus images. However, participants were still able to determine if the lesion was benign or malignant, which might warrant referral to specialist care.

## Figures and Tables

**Table 1:** Comparing diagnostic accuracy between simulated and non-simulated images for various types of choroidal lesions.

		Non-Simulated Images Diagnosis Score	Simulated Protanopia Images Diagnosis Score		Simulated Deuteranopia Images Diagnosis Score		Simulated Tritanopia Images Diagnosis Score	
Choroidal Lesion		Correct (%)	Correct (%)	*p* Value	Correct (%)	*p* Value	Correct (%)	*p* Value
Normal	Diagnosis	41 (100%)	32 (78%)	0.002	33 (81%)	0.005	40 (98%)	1.00
	Need for referral	41 (100%)	40 (98%)	1.00	40 (98%)	1.00	41 (100%)	1.00
Hemangioma	Diagnosis	16 (39%)	5 (12%)	0.01	6 (15%)	0.02	13 (32%)	0.64
	Need for referral	38 (93%)	31 (76%)	0.70	34 (83%)	0.31	35 (85%)	0.47
Nevus	Diagnosis	36 (88%)	26 (63%)	0.019	24 (59%)	0.005	34 (83%)	0.75
	Need for referral	39 (95%)	32 (78%)	0.007	30 (73%)	0.003	38 (93%)	1.00
Melanoma	Diagnosis	38 (93%)	30 (73%)	0.03	30 (73%)	0.03	36 (88%)	0.70
	Need for referral	41 (100%)	39 (95%)	0.49	40 (98%)	1.00	41 (100%)	1.00
Metastasis	Diagnosis	30 (73%)	19 (46%)	0.024	18 (44%)	0.013	26 (63%)	0.47
	Need for referral	38 (93%)	34 (83%)	0.31	35 (85%)	0.48	39 (95%)	1.00

Number of participants who correctly diagnosed the image, and number of those who decided correctly if this patient had a disease that needs referral or not. The correct answer for Melanoma and metastasis is that they need referral, while the correct answer for the others is that they do not need referral.

**Table 2:** Comparing accuracy in diagnosing simulated and non-simulated images between general ophthalmologists on one side and retina specialists and ocular oncologists on the other side.

41 Participants (41)		Specialty			
		Overall (41)	General Ophthalmologists (28)	Retina Specialist & Ocular Oncologist (13)	*p* Value
		N (%)	N (%)	N (%)	
Non-simulated images diagnosis score	Overall Score	36 (88%)	23 (82%)	13 (100%)	0.159
	Benign vs. Malignant	40 (98%)	27 (96%)	13 (100%)	1.00
	*p* value	0.201	0.19	1.00	
Simulated protanopia images diagnosis score	Overall Score	30 (%)	20 (%)	10 (%)	1.00
	Benign vs. Malignant	38 (%)	26 (%)	12 (%)	1.00
	*p* value	0.037	0.07	0.59	
Simulated deuteranopia images diagnosis score	Overall Score	28 (%)	18 (%)	10 (%)	0.49
	Benign vs. Malignant	37 (%)	25 (%)	12 (%)	0.45
	*p* value	0.027	0.055	0.59	
Simulated tritanopia images diagnosis Score	Overall Score	35 (%)	23 (%)	12 (%)	0.644
	Benign vs. Malignant	39 (%)	26 (%)	13 (%)	1.00
	*p* value	0.26	0.42	1.00	

## Data Availability

Data are available on reasonable request upon demand made to the corresponding authors.
